# Association Between Oregon’s 12-Month Contraceptive Supply Policy and Quantity of Contraceptives Dispensed

**DOI:** 10.1001/jamahealthforum.2021.5146

**Published:** 2022-02-18

**Authors:** Maria I. Rodriguez, Sunny C. Lin, Maria Steenland, K. John McConnell

**Affiliations:** 1 Department of Obstetrics and Gynecology, Oregon Health & Science University, Portland; 2 Center for Health Systems Effectiveness, Oregon Health & Science University, Portland; 3 OHSU-PSU School of Public Health, Portland, Oregon; 4 Population Studies and Training Center, Brown University, Providence, Rhode Island

## Abstract

**Importance:**

Eighteen states, including Oregon, have passed legislation requiring insurers to cover dispensation of a 12-month supply of short-acting, hormonal contraception.

**Objective:**

To determine whether Oregon’s 2016 12-month supply law was associated with an increase in contraceptive supply received.

**Design, Setting, and Participants:**

This retrospective cohort study of hormonal contraceptive users using Oregon’s All Payer All Claims database examined the quantity of contraceptive supply dispensed 3 years before and 3 years after the 2016 policy change. We also assessed changes among patients attributed to Title X clinics.

**Exposures:**

Legislation requiring insurers in Oregon to cover a 12-month supply of contraception to continuing users.

**Main Outcomes and Measures:**

Receipt of a 12-month supply of hormonal contraception

**Results:**

This cohort study of insured users (mean [SD] age, 27.4 [2.1] years) of short-acting hormonal contraception included 639 053 contraceptive prescriptions. Results indicated that more than 80% of prescriptions for contraceptives cover 3 months or fewer. Most women in the study population used the oral contraceptive pill, lived in a metropolitan area, and were privately insured. We did not observe a significant association between the passage of the 12-month supply policy and receipt of a 12-month supply (aOR, 1.01; 95% CI, 0.74-1.38). Receipt of a 12-month supply was more common for Medicaid recipients than the privately insured (aOR, 9.40; 95% CI, 6.62-13.34). We did find a shift from 1 month to 2 to 3 months supply being dispensed. The policy change was associated with a small, overall increase in quantity dispensed (0.27 months supply; 95% CI, 0.15 to –0.38). Title X clinics prescribed 3 months more of contraceptive supply than non–Title X clinics (3.03 months supply; 95% CI, 2.64-3.41). However, the policy change was not associated with increased contraceptive supply dispensed at Title X clinics.

**Conclusions and Relevance:**

In this cohort study of insured users of short-acting hormonal contraception, the passage of a 12-month contraceptive supply policy was not associated with an increase in contraceptive supply dispensed.

## Key Points

**Question:**

Is legislation requiring insurers to cover 12 months of short-acting hormonal contraception associated with an increase in 12-month contraceptive prescriptions?

**Findings:**

This cohort study found that more than 80% of prescriptions for contraceptives cover 3 months or fewer. Legislation mandating insurance coverage of annual prescriptions was not associated with an increase in 12-month prescriptions, although it was associated with a shift from 1-month prescriptions to prescriptions for 2 to 3 months.

**Meaning:**

Eliminating the need to return to a pharmacy every 30 or 90 days to refill prescriptions will likely require changes beyond insurance coverage and may necessitate outreach and education to clinicians and insurers.

## Teaser

This retrospective cohort study of hormonal contraceptive users examines the quantity of contraceptive supply dispensed 3 years before and 3 years after the 2016 policy change requiring insurers to cover dispensation of a 12-month supply of short-acting, hormonal contraception.

## Introduction

The ability to decide if or when to become pregnant is fundamental to individual rights, health, and people’s role in society.^1^ Contraception is a safe and highly effective intervention to prevent pregnancy. In the US, many women rely on the oral contraceptive pill to prevent pregnancy. With perfect use, it has 99% efficacy in preventing pregnancy.^2^ However, breaks in use are common, reducing the effectiveness of oral contraceptives.^2^

The Affordable Care Act eliminated cost-sharing for contraceptives. Twelve-month prescription policies are an essential next step to reduce barriers to contraceptive access.^3^ Contraceptive 12-month supply policies require insurers to cover the cost of dispensing a full 12 months of coverage per prescription.^4^ In the absence of such policies, clinicians can prescribe a 12-month supply, but insurance coverage typically dictates the amount a person receives.^5^ These policies thus focus on changing insurance company behavior to improve access.

The justification for 12-month supply policies is based on studies demonstrating that women are more likely to continue contraceptive use when dispensed at least a 6-month supply.^6,7^ Despite this evidence, a national survey in 2017 found that most women in the US (70%) receive a contraceptive supply of 3 months or less.^8^ Currently, 18 states have adopted legislation requiring insurers to cover the provision of 6 to 12 months of contraceptive supply at a time.^4^ For these policies to be effective, insurance companies would need to comply and be held accountable for following the revised coverage guidelines. Similarly, clinicians would need to change their standard prescribing patterns to write for an extended supply of contraception, and pharmacists would need to dispense the full supply. However, there have been little data to demonstrate whether these policies have been fully implemented and led to changes in prescribing practices.

On January 1, 2016, Oregon implemented legislation requiring all public and private insurers to cover the provision of 12 months of contraceptive coverage at once for all individuals renewing a prescription.^5^ Individuals who had not used the prescribed contraceptive method previously were to be dispensed a 3-month supply; after this 3-month trial, insurers would be required to cover a full year’s supply.^5^ The law covered all short-acting methods of contraception: the pill, patch, and ring.

Following the policy change, the state of Oregon’s Reproductive Health Program disseminated information on the policy and its evidence base to clinicians enrolled in the state’s network of 150 publicly funded contraceptive clinics. This network of publicly funded clinics consisted of sites receiving Medicaid and Title X funding. Title X clinics are community health centers that receive funds from the federal government specifically designated to provide comprehensive contraceptive counseling and services. Title X clinics provide care for people with no insurance, Medicaid, and private insurance. Title X clinics receive grant funds that allow them to cover the costs of services for uninsured people. It is possible that Title X funds were used to cover a 12-month supply prior to the policy change. Oregon’s Reproductive Health Program reimbursed for contraceptive claims from private clinicians and those in the Title X network. Oregon Medicaid covered the cost of a 12-month supply from the beginning of the policy change. It is not known how, or if, private insurance plans complied with the law. There were no incentives or penalties tied to the policy and we were unaware of any regulatory oversight performed to ensure implementation of the policy or accountability for adhering to the law. Some private insurance plans—including large national plans with a presence in Oregon—may not have been aware of the Oregon policy change. Furthermore, self-insured commercial plans were not subject to state law.^9^

We used Oregon’s All Payer All Claims database to analyze the effect of this policy change on the quantity of contraceptives prescribed and dispensed for publicly and privately insured women in Oregon. We hypothesized that the policy change would be associated with an increase in the supply of short-acting methods of contraception (pill, patch, ring) dispensed. We also assessed the policy’s effect for women who received contraceptives from Title X clinics. Given Title X clinics' emphasis on evidence-based reproductive health guidelines, we hypothesized that Title X clinics would be more likely to dispense prescriptions with a 12-month supply than non–Title X clinics.

## Methods

### Data Sources and Study Population

We conducted a retrospective cohort study of insured women in Oregon (ages 12 through 51 years) using short-acting forms of hormonal contraception between January 1, 2013, and December 31, 2018, allowing for 3 years of data before and after the January 1, 2016, policy change.^5^ The institutional review board at Oregon Health & Science University approved the study, which waived written informed consent because all data were deidentified. We analyzed data spanning May 1, 2021, to September 10, 2021. We followed the Strengthening the Reporting of Observational Studies in Epidemiology (STROBE) reporting guideline.

**Figure 1.**
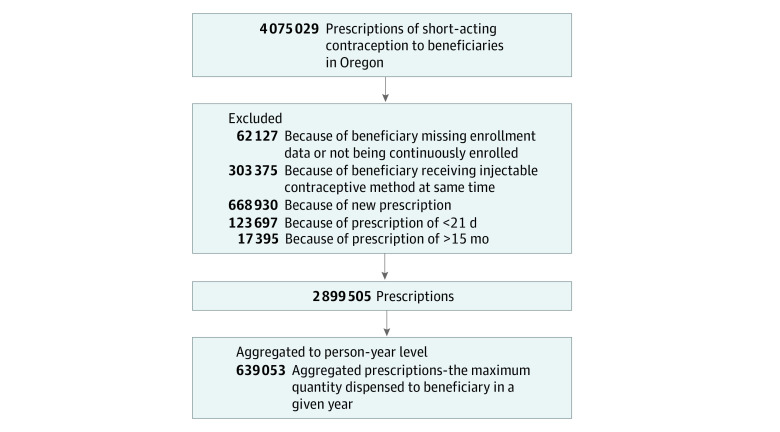
Study Cohort Creation: Prescriptions of Short-Acting Contraceptives, Oregon, 2013 to 2018

We obtained enrollment data and medical and pharmacy claims from the Oregon Health Authority. We used Medicaid claims for Medicaid enrollees and the Oregon All Payer All Claims (APAC) data for privately insured enrollees. The APAC database contains claims from the largest 25 commercial insurers in Oregon, excluding information from small carriers with fewer than 5000 enrollees and from certain federal programs such as the Federal Employee Health Benefits Program.^10^ We combined medical and pharmaceutical claims data for all beneficiaries who received between 21 (>1 pack) and 420 days (<15 packs) worth of short-acting contraceptives. We included individuals who had complete demographic data and were between 12 and 51 years of age when the prescription was dispensed (Figure 1). We conducted our analysis at the person-year level.

We excluded claims for prescriptions dispensed in quantities fewer than 21 days and more than 420 days (15 packs); claims of fewer than 21 days may be for noncontraceptive use, and claims of more than 420 days were rare (<1%). We also excluded claims for short-acting contraceptives if they occurred at the same time as a hormonal contraceptive injection or implant (Figure 1). We excluded claims for new prescriptions because the legislation limits dispensing quantity to a 3-month supply for individuals initiating a new contraceptive method for the first time.^5^ We defined new prescriptions as prescriptions for whom the person would have run out of their last prescription more than 30 days before their next prescription was filled, as well as the first prescription for each beneficiary in our data set.^11^

We matched the National Provider Identifier (NPI) of the prescriber with data from the National Plan & Provider Enumeration System (NPPES) to determine whether the prescribing clinician was part of a Title X clinic.^12^ We used organizational and health care professional NPIs to determine clinic Title X status and confirmed it was a Title X clinic by linking billing zip code with the Office of Population Affairs Title X Directory of clinics.^13^

### Dependent Variables

Our primary outcome was a binary variable indicating the receipt of a 12-month supply of contraceptives. To determine whether a prescription was dispensed as a 12-month supply, we calculated the number of months (ie, “packs”) that each dispensing quantity would have covered if taken at the recommended dose. To account for people who may be taking active oral contraceptives pills continuously (ie, skipping inactive pills), we defined 12-month supply as any prescription dispensed in quantities between 12 to 15 packs. We then aggregated the data to the beneficiary-year level by including only the claim for the largest quantity dispensed in each given calendar year. We prioritized the claim with the largest quantity dispensed in each calendar year to try and identify whether the policy change was associated with an increase in the maximum amount dispensed. In a secondary analysis, we repeated our models using month supply as a continuous variable, truncating the continuous variable at 12 months.

### Independent Variables

We included the following claim-level characteristics: payer (Medicaid or private insurance), age, rural residence (defined using Rural-Urban Commuting Area codes), and whether the prescriber was at a Title X clinic.^14^

### Statistical Analysis

We estimated 3 models. We conducted a logistic regression examining the association between the policy and our primary outcome: receipt of 12 months or more of contraception, controlling for payer type, age category, geographic area, and contraceptive type. Our second model used linear regression to estimate the association between the policy and the continuous measure of months of contraceptives prescribed, controlling for payer type, age category, geographic area, and contraceptive type. Finally, given Title X’s specialized funding and emphasis on reproductive health care, we conducted a subanalysis examining whether the policy had a differential effect on Title X clinics vs other prescribers. We ran a linear regression model that included an interaction term between the policy and whether the prescriber was a Title X clinic, controlling for payer type, age category, and geographic area. In all 3 models, we clustered standard errors at the prescriber level (by NPI).

We also conducted a sensitivity analysis where we examined the sensitivity of our findings to our definition of new prescription. We ran our models with 2 different definitions of new prescription to assess whether our findings would change with variations in the definition of new prescription. Our baseline definition of new prescription was that an individual had not used the method in at least 30 days. We reran the models changing the definition to 90 days (n = 558 293) and 180 days (n = 513 465); our results remained robust (eTable 2 in the Supplement).

We used Stata statistical software (version 16.1, Stata Corp) for all analyses.

## Results

Our final sample consisted of 639 053 prescriptions, among a population that was largely privately insured (71.8%) and in a metropolitan area (78.6%) (Table 1). Most prescriptions were for the oral contraceptive pill (90.8%). The number of individuals cared for by Title X clinics was relatively stable over time: 7.6% of women received care in a Title X clinic prepolicy compared with 6.4% postpolicy. Eighteen percent of Medicaid-enrolled women received their contraceptives from Title X clinics, compared with 2% of women with private insurance.

**Table 1.** Demographic Characteristics for Women in Oregon Who Received a 1 to 15 Month Supply of Short-Acting Contraception, 2013 to 2018 (N = 639 053)^a^

Characteristic	Beneficiaries, No. (%)		P value
	Prepolicy 2013-2015^b^	Postpolicy 2016-2018^b^	
No.	295 883	343 170	
Payer			
Medicaid	86 559 (29.3)	107 579 (31.4)	.005
Private	209 324 (71.8)	235 591 (69.7)	
Title X clinic			
No	273 291 (92.4)	321 394 (93.7)	.009
Yes	22 592 (7.6)	21 776 (6.4)	
Age, y			
12-17	29 640 (10.2)	33 075 (9.8)	.008
18-24	88 447 (30.4)	99 730 (30.0)	
25-34	104 313 (35.9)	122 367 (36.2)	
35-44	50 152 (17.3)	59 998 (17.8)	
45-51	18 242 (6.3)	22 797 (6.8)	
Geographic area			
Metropolitan	226 258 (78.6)	255 916 (76.8)	.009
Micropolitan	45 229 (15.7)	56 181 (16.9)	
Small town	9923 (3.5)	12 373 (3.7)	
Rural	6567 (2.3)	8596 (2.6)	
Contraceptive type			
Pill	268 673 (90.8)	309 621 (90.2)	.01
Vaginal ring	20 166 (6.8)	23 817 (6.9)	
Combined hormonal patch	7044 (2.4)	9732 (2.8)	

^a^ Short-acting contraception methods include the pill, ring, and patch without accompanying hormonal contraceptive injections.

^b^ Totals may not sum to 100% owing to rounding.

**Figure 2.**
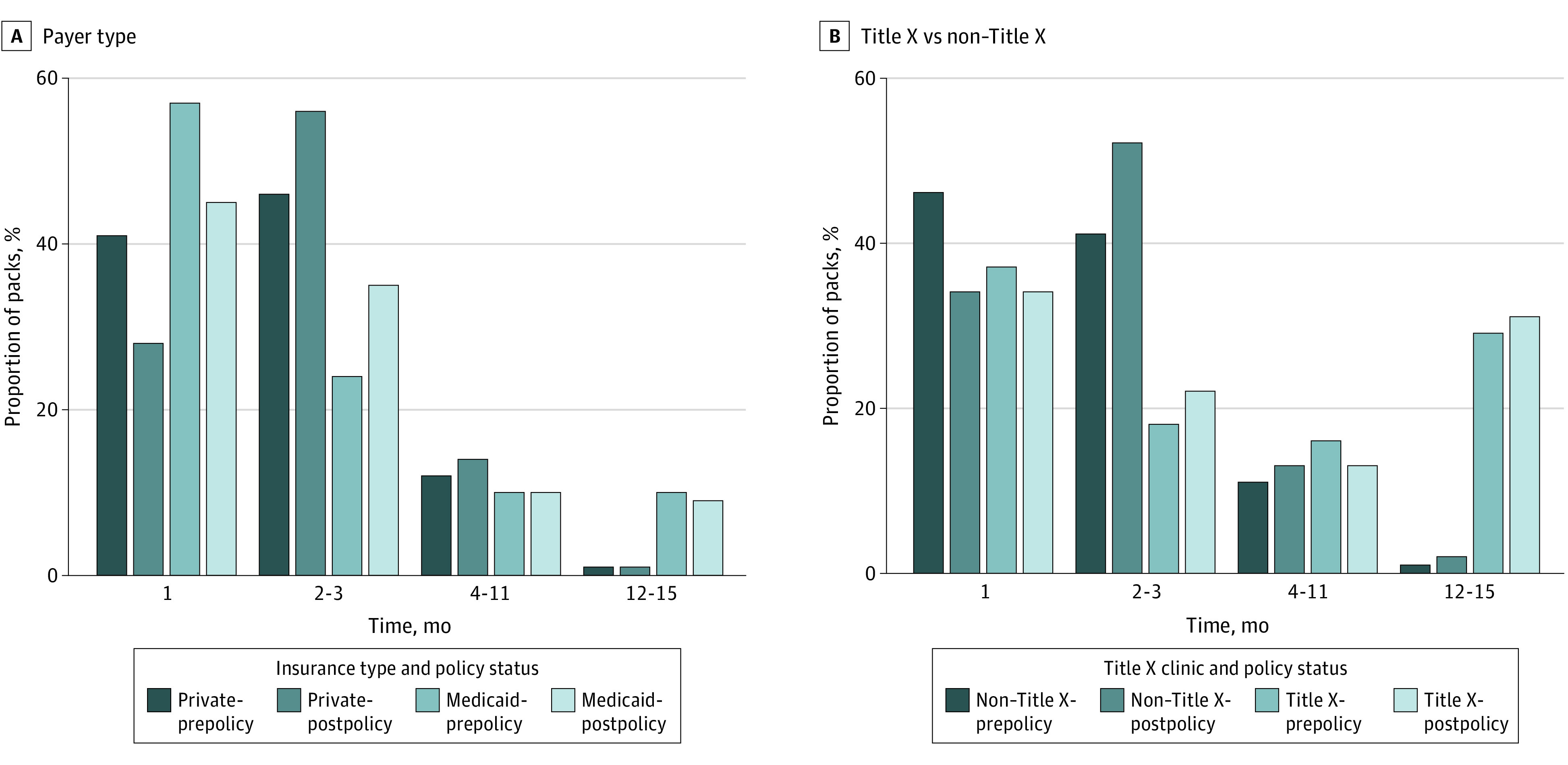
Months of Contraceptive Coverage Dispensed Over Time A, Proportion of packs dispensed by payer type before and after 12-month prescribing policy implementation, 2013 to 2018 (N = 611 045). B, Proportion of packs/months dispensed by Title X vs non–Title X clinics before and after 12-month prescribing policy implementation, 2013 to 2018 (N = 611 045).

Overall, only 3.5% of short-acting contraceptive users in Oregon received a 12-month supply prior to the policy change (eTable 1 in the Supplement). We did not observe a significant increase postpolicy in receipt of a 12-month supply (3.7%). However, there was a shift in the distribution of months prescribed. Prior to the policy change, most women received a prescription for between 1 to 3 months of coverage (80.7% for Medicaid; 86.7% for women with private insurance; eTable 1 in the Supplement; Figure 2A). Prepolicy, 57% of women with Medicaid received only 1 month of coverage, but this decreased to 45% after the policy change. The percent of women with Medicaid receiving 2 to 3 months of coverage increased from 24% to 35% (*P* < .001). The trend was similar for privately insured women. The share of women with private insurance receiving 1 month of coverage decreased from 41% to 28% (*P* < .001), while the share receiving 2 to 3 months of coverage increased from 46% to 56% (*P* < .001).

Prior to the policy change, women in Title X clinics were significantly less likely to receive a 1- to 3-month supply than women attending a non–Title X clinic (54.9% vs 87.6%; *P* < .001; eTable 1 in the Supplement; Figure 2B). They were also significantly more likely to receive a 12-month supply of contraception compared with women attending a non–Title X clinic before the policy change (28.6% vs 1.4%, *P* < .001).

Table 2 displays changes associated with the policy and receipt of a 12-month supply of short-acting contraceptives. We did not observe a significant association between the policy change and receipt of a 12-month supply (aOR, 1.01; 95% CI, 0.74-1.38; Table 2). The receipt of a 12-month supply was more likely among Medicaid recipients relative to individuals with private coverage (aOR, 9.42; 95% CI, 6.65-13.33). Compared to the youngest women in our sample (aged 12-17 years), women aged 18 to 24 years and women aged 25 to 34 years were more likely to receive a 12-month supply (aOR, 1.71; 95% CI, 1.55-1.88; and aOR, 1.36; 95% CI, 1.16-1.58) respectively, whereas women older than 45 years were less likely to receive a full year supply (aOR, 0.78; 95% CI, 0.64-0.96; Table 2).

**Table 2.** Association Between Oregon’s Contraceptive Supply Policy and Receipt of 12-Month Supply of Short-Acting Contraceptives Dispensed Among Oregon Beneficiaries, 2013 to 2018 (N = 611 045)^a^

Variable	Adjusted odds ratio (95% CI)^b^
Policy period	
Prepolicy	1 [Reference]
Postpolicy	1.01 (0.74-1.38)
Payer type	
Private	1 [Reference]
Medicaid	9.42 (6.65-13.33)
Medicaid payer × post-policy period	1.04 (0.75-1.44)
Age, y	
12-17	1 [Reference]
18-24	1.71 (1.55-1.88)
25-34	1.36 (1.17-1.58)
35-44	0.89 (0.75-1.05)
45-51	0.78 (0.64-0.96)
Geographic area	
Metropolitan	1 [Reference]
Micropolitan	0.59 (0.44-0.78)
Small town	0.63 (0.44-0.89)
Rural	0.69 (0.51-0.92)
Contraceptive type	
Pill	1 [Reference]
Patch	1.16 (0.84-1.58)
Ring	1.69 (1.50-1.90)

Abbreviation: NPI, National Provider Identifier.

^a^ For beneficiaries with 1 to 15 months of contraception dispensed. Contraceptive supplies between 13 to 15 months were truncated to 12 months.

^b^ Standard errors were clustered at the NPI level.

However, in analyses of contraceptive supply as a continuous outcome, we found a significant association between the policy change and quantity of contraceptive supply dispensed (0.27; 95% CI, 0.15-0.38; Table 3). Women enrolled in Medicaid were prescribed half a month more contraceptive coverage at a time compared with women enrolled in a private plan (0.47; 95% CI, 0.17-0.76). In addition, compared with women aged 12 to 17 years, women in all older age categories received a larger quantity of contraceptives at a time.

**Table 3.** Association Between Oregon’s Contraceptive Supply Policy and the Number of Months of Short-Acting Contraceptives Dispensed Among Beneficiaries, 2013 to 2018 (N = 611 045)^a^

Variable	Marginal effect (95% CI)^**b**^
Policy period	
Prepolicy	1 [Reference]
Postpolicy	0.27 (0.15 to 0.38)
Payer type	
Private	1 [Reference]
Medicaid	0.47 (0.17 to 0.76)
Medicaid × postpolicy	0.19 [−0.14 to 0.53]
Age, y	
12-17	1 [Reference]
18-24	0.33 (0.27 to 0.40)
25-34	0.21 (0.14 to 0.28)
35-44	0.20 (0.14 to 0.27)
45-51	0.29 (0.23 to 0.36)
Geographic area	
Metropolitan	1 [Reference]
Micropolitan	−0.43 (−0.58 to −0.28)
Small town	−0.46 (−0.66 to −0.25)
Rural	−0.44 (−0.60 to −0.29)
Contraceptive type	
Pill	1 [Reference]
Patch	2.06 (1.88 to 2.22)
Ring	0.18 (0.06 to 0.30)
Constant	2.26 (2.18 to 2.35)

Abbreviation: NPI, National Provider Identifier.

^a^ For beneficiaries with 1 to 15 months of contraception dispensed. Contraceptive supplies between 13 to 15 months were truncated to 12 months.

^b^ Standard errors were clustered at the NPI level.

We also examined the role that Title X clinics play in promoting access to a larger contraceptive supply. Overall, Title X clinics prescribed 3 months more of contraceptive supply than non–Title X clinics (3.03; 95% CI, 2.64-3.41; eTable 3 in the Supplement). However, the policy was not associated with a statistically significant change in quantity dispensed in Title X clinics relative to non–Title X clinics (−0.14; 95% CI, −0.65 to 0.37; eTable 3 in the Supplement).

## Discussion

We found that Oregon’s policy requiring insurers to cover 12 months of contraceptive supply was not associated with an increase in individuals receiving a 12-month supply. However, we did find a significant shift from women receiving only 1 month of coverage to being more likely to receive 2 or 3 months (Figure 2A). The increase we observed is equivalent to only 10 days supply more dispensed (0.27; 95% CI, 0.15-0.38). Importantly, most (>80%) women in Oregon were receiving a 3-month supply of contraception or less, even after the policy change.

We did find an exception to this pattern: approximately a third of women receiving contraception in a Title X clinic received a 12-month contraceptive supply. However, Title X clinics account for less than 8% of prescriptions in Oregon, indicating the importance of strengthening access to 12-month supply for individuals broadly in the state. Our findings demonstrate that women and adolescents continue to experience the burden of frequent pharmacy visits to maintain continuous contraceptive use and prevent unintended pregnancy. These findings suggest that 12-month policies alone, without additional efforts, are unlikely to provide women with the option of a longer-term supply.

However, we did find important differences in receiving a 12-month supply by important characteristics. The odds of receiving a 12-month supply was 9 times greater among individuals with Medicaid coverage than among individuals with private insurance coverage. These results likely reflect the baseline strength of Oregon’s publicly funded family planning network, which regularly implements evidence-based trainings and innovations in contraceptive service delivery, including dispensing an extended supply of short-acting methods.^15,16^

We also found that individuals who received care at a Title X clinic received 3 more months of contraception than those who received care in non–Title X clinics. Title X clinics, which receive financial support from the federal government, may be more familiar with best practices issued by the government agencies. For example, Title X clinics may have been aware of the 2014 Office of Population Affair’s guidelines for providing quality family planning services, which recommend providing a year-long supply.^17^ Title X clinics also dispense contraceptives on-site, which removes another barrier to accessing a 12-month supply.

There are several potential reasons why the policy was not enough to increase receipt of a 12-month supply of contraception. The first is that patients and clinicians may not have been aware of the law, so individuals may not have known that they could request it, and clinicians may not have offered a 12-month supply during counseling. In addition, pharmacists who were not aware of the law may have been unwilling to fill longer-term presciptions, as has been reported in other states with 12-month supply laws.^18,19^

Although Medicaid guaranteed coverage for a 12-month supply in Oregon, coverage by private plans for dispensing a 12-month supply may vary. The prevalence of self-insured private plans in Oregon may also partially explain our findings. Among Oregon’s privately insured population, 43% are covered by self-insured plans.^20^ In self-insured plans, the employer contracts to provide the employee health benefits and assumes the financial risk. These plans are regulated under the Employee and Retirement Income Security Act of 1974 (so-called ERISA plans) and are exempt from state law.^9^ Additional research is needed to better understand the degree to which these factors played in the minimal policy effect we observed.

Ongoing, low rates of 12-month supplies of oral contraception represent missed opportunities to promote reproductive autonomy. Previous research has documented that women who received a greater supply of oral contraception had increased continuous use and decreased rates of unintended pregnancy.^6,21,22^ As of 2020, 12-month supply laws have been enacted in 18 states (including Oregon),^23^ but limited evidence exists on whether they have been fully implemented. Additional research is needed to understand the effect of these policy changes on contraceptive supply and identify barriers to policy implementation.

### Limitations

This study has several limitations. Our study relied on administrative data; we did not have information on individual values or fertility preferences. We also lacked information on additional demographic and clinical variables that may have affected contraceptive use, such as pregnancy history. Some individuals might not want a 12-month supply—for example, if they want to become pregnant within the next year. However, the small share of individuals who received a 12-month supply, particularly for the privately insured (1%), suggests some unmet need for long-term contraceptive supply.^20^ Our use of claims data allowed us to see what was reimbursed by the insurer. We were unable to see the quantity that was initially prescribed and compare this to what was dispensed by the pharmacist or reimbursed by the insurer. However, in Oregon, Medicaid has fully reimbursed for a 12-month supply since the policy change. This indicates that for the 90.1% of Medicaid recipients who did not receive a 12-month supply following the policy change, that factors other than insurance coverage were responsible for the smaller supply.

Another limitation of our study is that we did not fully capture all self-insured individuals in our data set and could not differentiate between self-insured and private plans. A 2016 Supreme Court decision made it optional for self-insured plans to report data.^24^ To address this limitation, we examined enrollment in private and self-insured plans across our study period. We did not observe a decrease in enrollment following the Supreme Court decision suggesting that the self-insured population in Oregon’s APAC data remained stable (eTable 6 in the Supplement). Furthermore, our study focused on the initial years of the policy, and implementation efforts may have increased over time.

Finally, this study was based on data from a single state, which affects generalizability. Consistent with national trends, long-acting reversible contraceptive (LARC) use in Oregon increased over the study period, rising from 10% to 15% of all contraceptive users in Medicaid. This study focused on individuals choosing to use short-acting forms of contraception. However, increases in LARC use at the population level would be unlikely to affect the number of pill packs prescribed by a clinician.^25^

## Conclusions

This cohort study found that a law mandating a 12-month contraceptive supply coverage was not associated with an increase in prescriptions covering 12 months, and only a modest increase in total supply dispensed. Increasing prescriptions to encompass a 12-month supply will likely require broad outreach to patients, health care professionals, and payers, as well as enforcement from state governments. Federal policy mandating coverage of a 12-month supply is another strategy to support access for individuals using short-acting contraception.

## Notes

Supplement. eTable 1. Frequency of oral contraceptives dispensed by duration of supply 2013-2018 (n=639 053) eTable 2. Sensitivity Analyses Varying Time Period for Determining New Prescription: Association between Oregon’s contraceptive supply policy and a 12 montha supply of short-acting contraceptives dispensed among Oregon beneficiaries, 2013-2018, (n = 611 045) eTable 3. Sensitivity Analyses Varying Time Period for Determining New Prescription: association between Oregon’s contraceptive supply policy and the number of months of short-acting contraceptives dispensed among beneficiaries, 2013-2018 (n=611 045) eTable 4. Differential impact of contraceptive supply policy on the quantity of short-acting contraceptives dispenseda for individuals receiving contraception from Title X clinics vs non-Title X clinics, Oregon beneficiaries, 2013-2018 (n=611 045) eTable 5. Differential impact of contraceptive supply policy on the quantity of short-acting contraceptives dispensed for Medicaid enrollees receiving contraception from Title X clinics vs non-Title X clinics, Oregon beneficiaries, 2013-2018 (n=611 045) eTable 6. Number of privately insured contraceptive users in Oregon by study year
