# Supplementary material for: Association Between Oregon’s 12-Month Contraceptive Supply Policy and Quantity of Contraceptives Dispensed
Source: JAMA Health Forum. 2022 Feb 18;3(2):e215146. doi: 10.1001/jamahealthforum.2021.5146 (PMC8903112; doi:10.1001/jamahealthforum.2021.5146)
Supplement: Supplement. — eTable 1. Frequency of oral contraceptives dispensed by duration of supply 2013-2018 (n=639 053) eTable 2. Sensitivity Analyses Varying Time Period for Determining New Prescription: Association between Oregon’s contraceptive supply policy and a 12 montha supply of short-acting contraceptives dispensed among Oregon beneficiaries, 2013-2018, (n = 611 045) eTable 3. Sensitivity Analyses Varying Time Period for Determining New Prescription: association between Oregon’s contraceptive supply policy and the number of months of short-acting contraceptives dispensed among beneficiaries, 2013-2018 (n=611 045) eTable 4. Differential impact of contraceptive supply policy on the quantity of short-acting contraceptives dispenseda for individuals receiving contraception from Title X clinics vs non-Title X clinics, Oregon beneficiaries, 2013-2018 (n=611 045) eTable 5. Differential impact of contraceptive supply policy on the quantity of short-acting contraceptives dispensed for Medicaid enrollees receiving contraception from Title X clinics vs non-Title X clinics, Oregon beneficiaries, 2013-2018 (n=611 045) eTable 6. Number of privately insured contraceptive users in Oregon by study year [file jamahealthforum-e215146-s001.pdf]

Supplemental Online Content

Rodriguez MI, Lin SC, Steenland M, McConnell K.J. Association between Oregon's 12-month contraceptive supply policy and quantity of contraceptives dispensed. *JAMA Health Forum*. 2022;3(2):e215146. doi:10.1001/jamahealthforum.2021.5146

eTable 1. Frequency of oral contraceptives dispensed by duration of supply 2013-2018 (n=639 053)
eTable 2. Sensitivity Analyses Varying Time Period for Determining New Prescription: Association between Oregon's contraceptive supply policy and a 12 montha supply of short-acting contraceptives dispensed among Oregon beneficiaries, 2013-2018, (n = 611 045)
eTable 3. Sensitivity Analyses Varying Time Period for Determining New Prescription: association between Oregon's contraceptive supply policy and the number of months of short-acting contraceptives dispensed among beneficiaries, 2013-2018 (n=611 045)
eTable 4. Differential impact of contraceptive supply policy on the quantity of short-acting contraceptives dispenseda for individuals receiving contraception from Title X clinics vs non-Title X clinics, Oregon beneficiaries, 2013-2018 (n=611 045)
eTable 5. Differential impact of contraceptive supply policy on the quantity of short-acting contraceptives dispensed for Medicaid enrollees receiving contraception from Title X clinics vs non-Title X clinics, Oregon beneficiaries, 2013-2018 (n=611 045)
e-Table 6. Number of privately insured contraceptive users in Oregon by study year

This supplemental material has been provided by the authors to give readers additional information about their work.

© 2022 Rodriguez MI et al. *JAMA Health Forum.*

eTable 1. Frequency of oral contraceptives dispensed by duration of supply 2013-2018 (n=639 053)

Number of packs	Type of insurance payor or clinic funding source, No. (%)							
	Medicaid		Private Insurance		Title X		Non-Title X	
	**Pre-policy** 86 559	**Post-policy** 107 579	**Pre-policy** 209 324	**Post-policy** 235 591	**Pre-policy** 22 592	**Post-policy** 21 776	**Pre-policy** 273 291	**Post-policy** 321394
1 packs	48 995 (56.6)	48 616 (45.2)	85 468 (40.8)	66 565 (28.3)	8 289 (36.7)	7 345 (33.7)	126 174 (46.2)	107 836 (33.6)
2-3 packs	20 868 (24.1)	37 917 (35.2)	96 452 (46.1)	132 520 (56.3)	4 115 (18.2)	4 866 (22.3)	113 205 (41.4)	165 571 (51.5)
4-6 packs	6 152 (7.1)	8 259 (7.7)	21 787 (10.4)	31 107 (13.2)	2 200 (9.7)	1 634 (7.5)	25 739 (9.4)	37 732 (11.7)
7-9 packs	1 377 (1.6)	2 116 (2.0)	3 282 (1.6)	2 169 (0.9)	820 (3.6)	729 (3.3)	3 839 (1.4)	3 556 (1.1)
10-11 packs	928 (1.1)	899 (0.8)	327 (0.2)	435 (0.2)	702 (3.1)	469 (2.2)	553 (0.2)	865 (0.3)
12-15 packs	8 239 (9.5)	9 772 (9.1)	2 008 (1.0)	2 795 (1.2)	6 466 (28.6)	6 733 (30.9)	3 781 (1.4)	5 834 (1.8)

© 2022 Rodriguez MI et al. *JAMA Health Forum.*

eTable 2. Sensitivity Analyses Varying Time Period for Determining New Prescription: Association between Oregon's contraceptive supply policy and a 12 month[a] supply of short-acting contraceptives dispensed among Oregon beneficiaries, 2013-2018, (n = 611 045)

	New prescription = 30 days (main analysis)	New prescription = 90 days	New prescription = 180 days
Post-policy period (Ref: Pre-policy)	0.95	0.96	0.95
Medicaid payer type (Ref: Private)	9.41***	9.10***	9.03***
Age (Ref: 12-17)			
18-24	1.72***	1.72***	1.70***
25-34	1.36***	1.36***	1.35***
35-44	0.89	0.89	0.880
45-51	0.78*	0.79*	0.788*
Geographic area (Ref: Metropolitan)			
Micropolitan	0.59***	0.60***	0.60***
Small town	0.63***	0.63*	0.63**
Rural	0.69*	0.70*	0.70*
Type (Ref: Pill)			
Patch	1.16	1.16	1.14
Ring	1.68***	1.72***	0.74***

Abbreviations: Ref, Reference

*p<0.05, **p<0.01, ***p<0.001

© 2022 Rodriguez MI et al. *JAMA Health Forum.*

eTable 3. Sensitivity Analyses Varying Time Period for Determining New Prescription: association between Oregon's contraceptive supply policy and the number of months of short-acting contraceptives dispensed among beneficiaries, 2013-2018 (n=611 045)[a]

	New prescription = 30 days (main analysis)	New prescription = 90 days	New prescription = 180 days
Post-policy period (Ref: Pre-policy)	-0.10	-0.11	-0.11
Medicaid payer type (Ref: Private)	0.46**	0.47**	0.48**
Age (Ref: 12-17)			
18-24	0.33***	0.34***	0.34***
25-34	0.21***	0.21***	0.21***
35-44	0.20***	0.19***	0.18***
45-51	0.29***	0.28***	0.28***
Geographic area (Ref: Metropolitan)			
Micropolitan	-0.43***	-0.43***	-0.43***
Small town	-0.46***	-0.45***	-0.45***
Rural	-0.44***	-0.43***	-0.43***
Type (Ref: Pill)			
Patch	2.06***	2.05***	2.04***
Ring	0.18**	0.20**	0.21**
Constant	2.14***	2.15***	2.16***

Abbreviations: Ref, Reference
*p<0.05, **p<0.01, ***p<0.001

© 2022 Rodriguez MI et al. *JAMA Health Forum.*

e-Table 4. Differential impact of contraceptive supply policy on the quantity of short-acting contraceptives dispensed[a] for individuals receiving contraception from Title X clinics vs non-Title X clinics, Oregon beneficiaries, 2013-2018 (n=611 045)

**Variable**	**Marginal Effect (95%CI)[b]**
Title X Clinic Pre Policy vs Not Title X Pre Policy	3.04 (2.65,3.43)
Non Title X Post Policy vs Non Title X Pre Policy	0.33 (0.28,0.37)
Title X Post Policy vs Title X Pre Policy	-0.17 (-0.68,0.34)
Medicaid payer type (Ref: Private)	-0.02 (-0.16,0.12)
Age (Ref: 12-17)	
18-24	0.26 (0.21,0.31)
25-34	0.18 (0.13,0.24)
35-44	0.23 (0.17,0.29)
45-51	0.33 (0.27,0.39)
Geographic area (Ref: Metropolitan)	
Micropolitan	-0.38 (-0.47,-0.29)
Small town	-0.42 (-0.58,-0.27)
Rural	-0.43 (-0.54,-0.31)
Type (Ref: Pill)	
Patch	2.15 (2.02,2.27)
Ring	0.14 (0.04,0.24)
Constant	2.19 (2.12,2.26)

Abbreviations: Ref, Reference; NPI, National Provider Identifier

[a]For beneficiaries with 1-15 months of contraception dispensed. Contraceptive supplies between 13-15 months were truncated to 12 months

[b]Standard errors were clustered at the NPI level

© 2022 Rodriguez MI et al. *JAMA Health Forum.*

e-Table 5. Differential impact of contraceptive supply policy on the quantity of short-acting contraceptives dispensed for Medicaid enrollees receiving contraception from Title X clinics vs non-Title X clinics, Oregon beneficiaries, 2013-2018 (n=611 045)

**Variable**	**Marginal Effect (95%CI)[b]**
Title X (Ref: Non-Title X)	3.72*** [3.34,4.09]
Post-Policy (Ref: Pre-Policy)	0.45*** [0.29,0.61]
Title X x Post Policy Interaction	-0.16 [-0.61,0.30]
Age (Ref: 12-17)	
18-24	0.35*** [0.27,0.43]
25-34	0.29*** [0.20,0.39]
35-44	0.10* [0.00,0.20]
45-51	0.08 [-0.04,0.20]
Geographic area	
(Ref: Metropolitan)	
Micropolitan	-0.40*** [-0.58,-0.22]
Small town	-0.43*** [-0.68,-0.19]
Rural	-0.41*** [-0.61,-0.21]
Type (Ref: Pill)	
Patch	1.54*** [1.40,1.68]

e-Table 6
Number of privately insured contraceptive users in Oregon by study year

Year	2013	2014	2015	2016	2017	2018	Total
Enrollees	72,527	66,085	70,712	75,688	81,107	78,796	444,915

© 2022 Rodriguez MI et al. *JAMA Health Forum.*
